# Estimating the decay of protective antibodies induced by SARS-CoV-2 mRNA vaccination and hybrid immunity

**DOI:** 10.1038/s44298-025-00156-3

**Published:** 2025-10-29

**Authors:** McKenna D. Roe, Si’Ana A. Coggins, Emily S. Darcey, Emilie Goguet, Hannah Haines-Hull, Dominic Esposito, Cara H. Olsen, Simon D. Pollett, Edward Mitre, Eric D. Laing

**Affiliations:** 1 Department of Microbiology and Immunology, Uniformed Services University, Bethesda, MD USA; 2 Henry M. Jackson Foundation for the Advancement of Military Medicine, Inc., Bethesda, MD USA; 3 Protein Expression Laboratory, Frederick National Laboratory for Cancer Research, Frederick, MD USA; 4 Department of Preventive Medicine and Biostatistics, Uniformed Services University of the Health Sciences, Bethesda, MD USA; 5 Infectious Diseases Clinical Research Program, Department of Preventive Medicine and Biostatistics, Uniformed Services University of the Health Sciences, Bethesda, MD USA

**Keywords:** RNA vaccines, SARS-CoV-2

## Abstract

Starting in 2020, we quantified anti-wildtype SARS-CoV-2 spike sera IgG at monthly intervals from generally healthy adults after various doses of mRNA-based COVID-19 vaccination and in the context of hybrid immunity. Confirmed post-vaccination infections and subclinical infections identified by longitudinal serology were removed from vaccine-only analyses. Over 400 days, the two-dose vaccine-alone antibody response decayed at a half-life (*t*_1/2_) of 59.8 days compared with a *t*_1/2_ of 99.7 days after receipt of one booster dose. In the hybrid immunity model, the *t*_1/2_ was greater at 241 days. Using cut-offs for correlation of protection obtained in a prior study, we modeled that individuals with hybrid immunity maintain antibody levels above a 75% correlate of immunity for 283 days after an immune-boosting event. These data offer insights into SARS-CoV-2 antibody decay kinetics that may inform COVID-19 vaccine timing in the younger (age under 60), healthy adult population.

## Introduction

The mRNA-based COVID-19 vaccines are effective at reducing risk for emergency department presentations, post-COVID-19 complications, and Long COVID^1–4^. Booster immunizations of SARS-CoV-2 (SC2) wildtype (WT) monovalent mRNA vaccines were associated with decreased instances of symptomatic infection and hospitalization compared to individuals who had only received the two doses of the primary vaccination series^5^. Booster immunizations are also known to increase the magnitude of detectable anti-SC2 antibodies and breadth of binding antibodies against Omicron variants (OMV)^6,7^. High levels of serum anti-SC2 WT spike IgG correlated with protection against post-vaccination-infection (PVI) during the 2021 OMV wave, but antibodies declined more rapidly than expected following immunization^8^. The kinetics of antibody responses have been reported by a variety of different studies but are often limited by low sample sizes and infrequent sampling^9–15^. The specific period of time following an immune boosting event (vaccination, infection, or both) that individuals remain protected from subsequent clinical infections remains speculative, in part due to lack of robust longitudinal serological data. This challenge has been compounded by the limitations in extrapolating immunological data measured across a range of ages to younger healthy adult populations for whom the evidence to support ongoing annual vaccine boosting remains less clear than for higher risk adults.

In this study, we conducted in-depth, prospective, longitudinal analyses of anti-SC2 WT IgG monthly for 400 days following an immune boosting event in younger healthy adults to provide a precise estimate of antibody decay following primary two-dose immunization with the Pfizer-BioNTech BNT162b2 mRNA vaccine, boosting with an mRNA WT monovalent booster, and infection following immunization, i.e., hybrid immunity. Further, we utilized previously reported correlate of protection thresholds at which individuals may remain 90%, 75%, and 50% protected from a PVI^16^ to estimate days post-infection/vaccination that an individual could remain protected from a PVI. While focused on an earlier pandemic period before 2022, these objectives have relevance to current vaccination booster schedule decision making, particularly for younger healthy adult populations.

## Results

### Study participant’s demographics

A total of 254 participants in the PASS cohort received two doses of the Pfizer-BioNTech BNT162b2 mRNA vaccine. Of these, 26 participants were excluded based on positive PCR or antigen test or IgG serology-confirmed PVI. An additional six participants were excluded due to lack of one-month post-vaccination sample collection (Supplementary Fig. 1). Detailed demographics of study participants have been previously described^16^. Of the 222 participants included in this study, 153 (69.0%) were women, 67 (30.0%) men, and 2 (1.0%) unreported. The median age was 41 [range 20–69; interquartile range 32–51].

### A third mRNA vaccine dose improves magnitude and durability of SARS-CoV-2 binding IgG

**Fig. 1:**
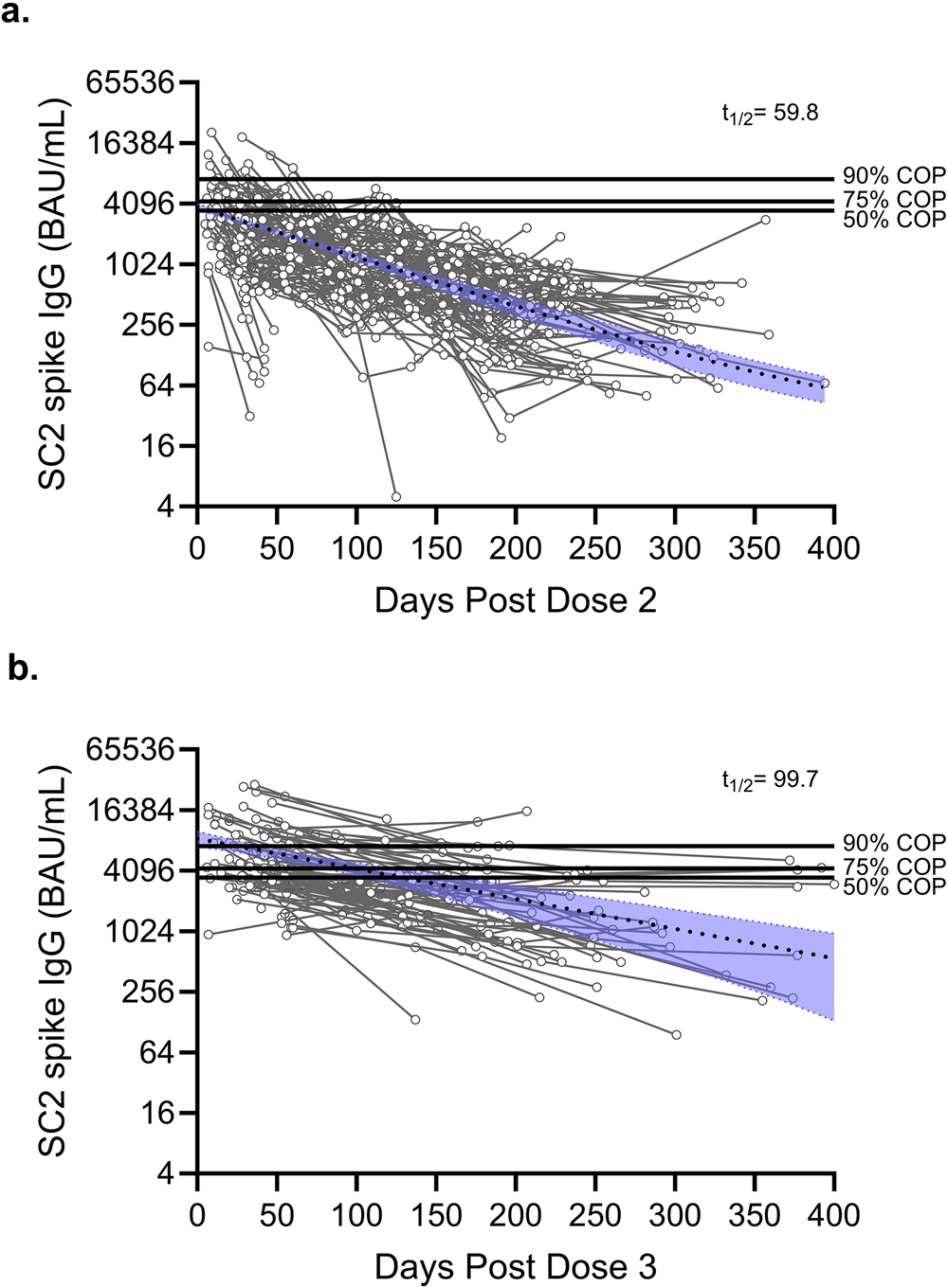
Vaccine-induced SARS-CoV-2 IgG durability improves after three doses. Serum samples from participants after receipt of (**a**) two BNT162b2 doses (*n* = 221) and (**b**) an mRNA booster immunization (*n* = 113) were tested for IgG against SARS-CoV-2 (SC2) WT spike in an antigen-based multiplex immunoassay. Half-life (*t*_1/2_) was calculated using a one phase decay curve model (dotted line) with a plateau constraint at an IgG level associated with a serostatus threshold cutoff in this test, 14.4 BAU/mL (WHO Binding Antibody Units). The 95% confidence intervals are shown in the blue shaded areas. Horizontal black lines at 3500 BAU/mL (50% COP), 4300 BAU/mL (75% COP), and 7200 BAU/mL (90% COP) represent antibody thresholds previously reported as protective percentages during the first Omicron BA.1, BA.1.1 wave^16^. COP correlate of protection.

To assess the rate of vaccine-induced antibody decay after a primary two-dose immunization series and a third WT-monovalent immunization (booster), we quantified anti-SC2 spike WT IgG at monthly intervals. The anti-SC2 spike IgG decay was estimated as the antibody half-life, or time taken for antibody levels to drop by 50% (*t*_1/2_). One- and two-phase decay models yielded similar fits (Supplementary Table 1), therefore a one-phase decay with least squares regression model was applied to IgG data collected over a 400-day interval based on the observed trends in IgG decay. One month following receipt of two vaccine doses, the mean anti-WT spike IgG levels were 2294 BAU/mL, which waned over 400 days with a *t*_1/2_ of 59.8 days [95% CI: 53.2–67.4] (Fig. 1a, Table 1). The magnitude of anti-WT spike IgG concentrations one month following receipt of a booster (third) vaccine dose was significantly higher, measured at 7199 BAU/mL (*p* < 0.0001) (Supplementary Fig. 2). Additionally, the IgG responses after the booster dose remained significantly more durable over 400 days with a *t*_1/2_ of 99.7 days [95% CI: 71.0–146.9], (F(1, 1447) = 15.5, *P* < 0.001) (Fig. 1a, b, Table 1).

**Table 1:** One phase decay model statistics following two doses of Pfizer-BioNTech BNT162b2 mRNA vaccine and one boost of a WT monovalent mRNA vaccine

Statistic	Post-two dose decay model^1^	Post-three dose decay model^2^
Y_0_ [95% CI]^3^	3811 BAU/mL [3525–4113]	8563 BAU/mL [7125–10,188]
Half-life [95% CI]	59.8 days [53.2–67.4]	99.7 days [71.0–146.9]
Time above 90% COP^4^	0 days	28 days
Time above 75% COP	0 days	99 days
Time above 50% COP	7 days	128 days

^1^Two dose primary immunization series was Pfizer-BioNTech BNT162b2 only. ^2^Pfizer (n = 112) and Moderna (n = 1)-manufactured vaccines included for post-three dose model. ^3^Y0 = Y-axis intercept. ^4^COP = correlate of protection, sera binding IgG concentrations as a percentage of protection previously reported^16^.

Previously, we calculated anti-SC2 spike IgG BAU/mL levels that were associated with protection from PVI during the OMV BA.1 wave that occurred from Dec 2021 to Feb 2022^16^. Within that study, anti-WT spike IgG concentrations of 7200, 4300, and 3500 BAU/ml correlated with protection levels of 90%, 75%, and 50%, respectively, against symptomatic PVI from OMV BA.1 and BA.1.1. Based on these antibody cut-offs, individuals who received two doses had circulating serum IgG above 50% BA.1/BA.1.1 protective levels for an average of seven days and did not have average antibody levels that reached the 75% or 90% protection thresholds. In contrast, individuals who received a booster immunization for a total of three doses had circulating IgG above 90%, 75%, and 50% protective levels for 28, 99, and 129 days, respectively (Table 1, Fig. 1a, b).

### Hybrid immunity improves durability of SARS-CoV-2 binding IgG

**Fig. 2:**
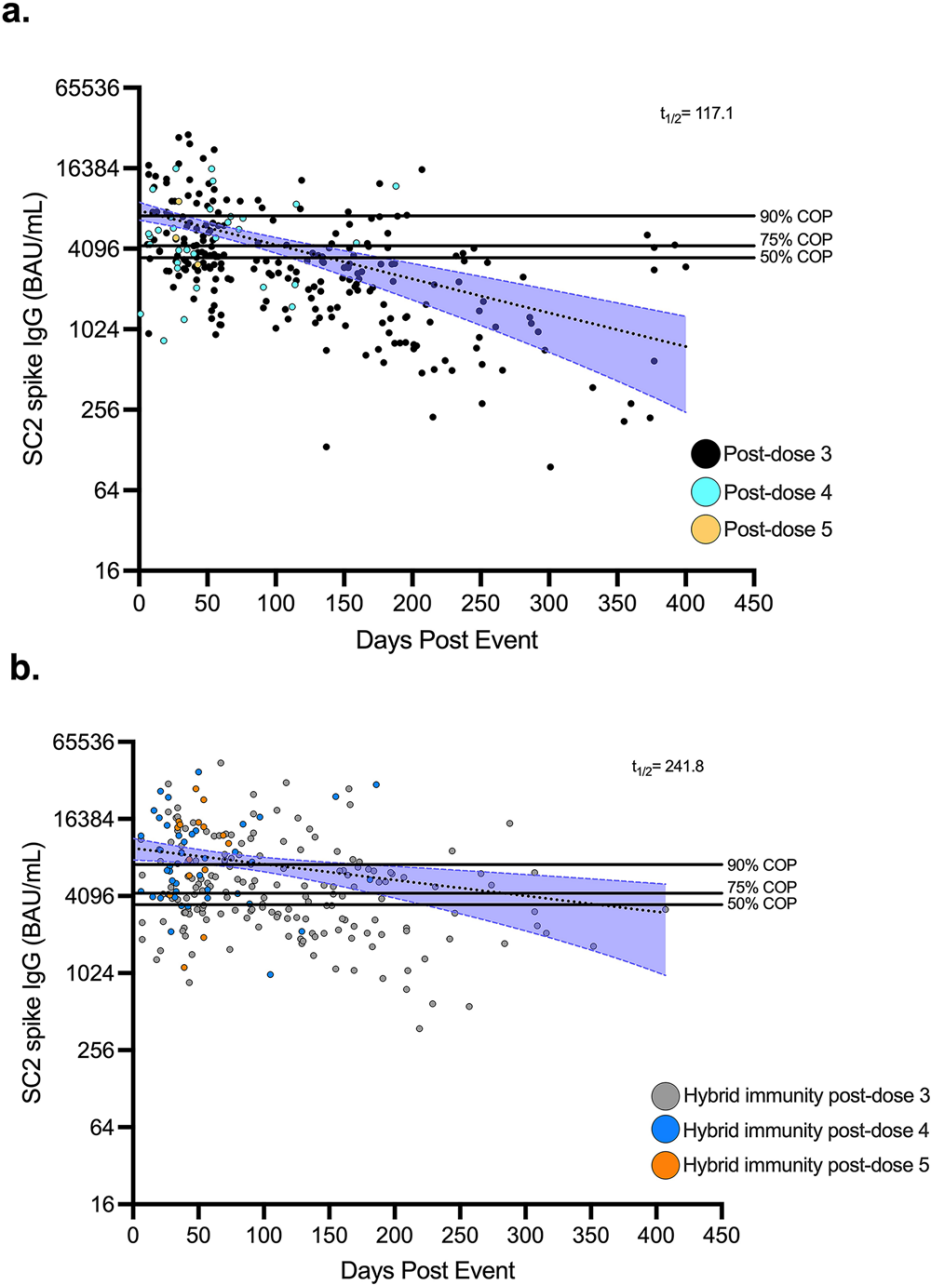
Anti-SC2 spike IgG durability in booster-only and hybrid immunity models. Serum samples from participants with (**a**) booster-only (*n* = 142) and (**b**) hybrid immunity (*n* = 160) were tested for IgG against SC2 spike. Anti-SC2 spike IgG was plotted as days after an immune boosting event, defined as either a SC2 post-vaccine infection or receipt of a third, fourth, and/or fifth booster dose. Serum samples are classified according to the number of booster doses received. A one phase decay curve model was fit to the data, shown by a dotted line with 95% confidence intervals in the shaded blue area. Horizontal black lines at 3500 BAU/mL (50% COP), 4300 BAU/mL (75% COP), and 7200 BAU/mL (90% COP) represent antibody thresholds previously reported as protective percentages during the first Omicron BA.1, BA.1.1 wave^16^. COP correlate of protection. BAU/mL, WHO Binding Antibody Units.

Next, we sought to examine the rate of antibody decay in the setting of hybrid immunity across the cohort, defined as a minimum of at least one booster dose and one or more SC2 infections, and then compared the kinetics to vaccine-alone immunity. The booster-only immunity IgG model incorporated anti-SC2 spike IgG responses from individuals with one or more booster doses and no prior evidence of PCR-confirmed or subclinical PVI. Anti-SC2 spike IgG decay in this group had a *t*_1/2_ of 117.1 days [95% CI: 84.1–173.0], and IgG levels were above 90%, 75%, and 50% protection cut-offs for 16, 103, and 138 days, respectively (Fig. 2a, Table 2). In comparison both the magnitude (Supplementary Fig. 3, *P* < 0.01) and decay of IgG (F(1, 495) = 3.89, *P* = 0.049) were both significantly greater in the hybrid immunity model compared to the booster-only group. The hybrid immunity IgG kinetics model estimated a *t*_1/2_ of 241.8 days [95% CI: 137.8–665.3], with respective IgG levels above 90%, 75%, and 50% BA.1/BA.1.1 protection for 103, 283, and 356 days (Fig. 2b, Table 2).

**Table 2:** One phase decay statistics for booster-only and hybrid immunity models

Statistic	Booster-only decay model^1^	Hybrid immunity decay model^2^
Y_0_ [95% CI]	7909 BAU/mL [6732–9202]	9677 BAU/mL [7950–11,605]
Half-life [95% CI]	117.1 days [84.1–173.0]	241.8 days [137.8–665.3]
Time at 90% COP	16 days	103 days
Time at 75% COP	103 days	283 days
Time at 50% COP	138 days	356 days

^1^Booster-only model consisted of post-third dose (*n* = 113), post-fourth dose (*n* = 26), and post-fifth dose (*n* = 3) serum samples. ^2^Hybrid immunity model consisted of post-third dose (*n* = 103), post-fourth dose (*n* = 42), and post-fifth dose (*n* = 15) serum samples with evidence of previous SARS-CoV-2 PVI.

## Discussion

In this study, we longitudinally examined the kinetics of anti-SC2 antibody decay post-mRNA immunization and following PVI using monthly serum samples collected from a well-characterized cohort of generally healthy adult healthcare workers in which clinical and subclinical infections were carefully measured. Consistent with prior studies, we observed antibody decay following two doses of the Pfizer-BioNTech mRNA vaccine to be ~2 months, which increases to ~3.25 months following receipt of one mRNA booster^9,10^. Interestingly, incorporation of individuals with more than one booster vaccination, i.e., four and five vaccinations, did not significantly alter the modeled SC2 spike IgG half-life (F(1, 496) = 0.398, *P* = 0.53) (Fig. 2a compared to Fig. 1b). Whereas, SARS-CoV-2 antibody durability in individuals with hybrid immunity exhibited more than double the half-life (~8 months) after an immune boosting event compared to the booster-only model. Although, in all models the antibody levels were predicted to decay below protective levels (50%) by one year.

Two doses of the Pfizer-BioNTech mRNA vaccine have been shown to effectively protect individuals from severe COVID-19, however effectiveness in protection against symptomatic PVI declines significantly after six months^12^. In this study, we evaluated antibody durability in the context of binding-antibody correlate of protection cutoffs that limited PVI^16^. We found that antibody levels remained above a 50% BA.1/BA.1.1 correlate of immunity level for 7 days after two vaccine doses and 128 days after three vaccine doses in the absence of prior clinical or subclinical infections. In contrast, individuals with hybrid immunity-maintained antibody levels above a 75% correlate of immunity level for 283 days after an immune-boosting event, a model most representative of the general younger healthy adult population who have experienced at least one SARS-CoV-2 infection.

The principal limitation of this approach was that we used thresholds against OMV BA.1, BA.1.1 and a WT vaccine background, and not up-to-date vaccine or circulating variants. The generalizability of estimated days of protection against BA.1/BA.1.1 PVI to currently circulating variants requires further investigation. Of note, the antigenic difference between KP.3 (the predominant circulating strain in November 2024) and JN.1 (the vaccine strain used in both mRNA COVID vaccines in the fall of 2024) is closer than the antigenic difference between OMV BA.1 and WT^17^, suggesting that this decay model is likely generalizable as long as the antigenic difference between the most recent booster vaccine variant and the predominant circulating strain are not markedly greater than the antigenic difference between WT and OMV BA.1. Additionally, we would have been under-powered to develop robust models had we attempted to further stratify individuals by booster vaccine type, infection-variant type, quantity of infections, or number of vaccine doses. Therefore, the impact of variant type, number of doses, and infection timing on antibody durability in the hybrid immune model is unknown and a limitation of this study.

Our data suggests that antibody levels may plateau to an upper concentration after multiple boosters and/or infections, potentially indicating that boosting before sufficient decay occurs may offer little to no increase in protective binding antibody levels. The impact of multiple booster doses on protective T cells responses and neutralizing antibodies was not assessed. Though, previously we demonstrated that anti-SARS-CoV-2 spike IgG binding antibodies reported in the WHO BAU were a correlate of protection and concordant with neutralizing antibody titers^16^. Nevertheless, our estimated hybrid immunity post-booster decay rates – which predict antibody waning to below protective levels between 6 and 12 months after last antigenic exposure – do provide some immunological rationale to consider annual boosting frequencies in healthy younger U.S. adults who have experienced at least one infection. These findings, alongside ongoing prospective vaccine clinical effectiveness and immunological assessment studies, may help support ongoing vaccine schedule decision making and public health messaging.

## Methods

### Study participants

Participants were enrolled in the Prospective Assessment of SARS-CoV-2 Seroconversion (PASS) study, a longitudinal, observational study initiated in August 2020, aimed to investigate the clinical and immunological responses to SARS-CoV-2 infection and vaccination^18^. This study protocol was approved by the Uniformed Services University of the Health Sciences Institutional Review Board (FWA 00001628; Department of Defense Assurance P60001) in compliance with all applicable Federal regulations governing the protection of human participants. All participants provided written informed consent for participation. The PASS cohort consists of generally healthy, adult (≥18 years old) healthcare workers who were employed at Walter Reed National Military Medical Center and were seronegative for SARS-CoV-2 with no history of COVID-19 diagnosis at the time of study enrollment. The subset of PASS participants included for analysis in this study also met the following criteria by December 17, 2021: (1) Fully vaccinated with two doses of the Pfizer-BioNTech BNT162b2 mRNA vaccine, (2) No history of COVID-19 diagnosis, SARS-CoV-2 infection, or anti-spike IgG seropositivity prior to vaccination with BNT162b2, (3) Provided serum samples corresponding to at least one month (20–50 days, 33 ± 7.8 days) post-full vaccination or receipt of the second BNT162b2 dose.

### Multiplex immunoassay

A quantitative microsphere-based multiplex immunoassay was established to test serum samples collected at monthly intervals against the pre-fusion stabilized spike proteins of WT SARS-CoV-2 and seasonal human coronaviruses HKU1, OC43, NL63, and 229E, and SARS-CoV-2 nucleoprotein using Luminex xMAP-technology as has been previously described^19,20^. Briefly, human serum samples were tested at four dilutions and anti-spike and anti-nucleoprotein IgG were measured as a median fluorescence intensity and interpolated against an internal reference standard. For anti-SC2 WT spike and nucleoprotein IgG, the reference standard was calibrated to the U.S. SARS-CoV-2 Serology Standard [NCI, Frederick National Laboratory] to obtain WHO binding antibody units (BAU/mL).

### Diagnosis of SARS-CoV-2 PVI

Participant data was classified by number of mRNA booster doses received and PVI history. Clinically-overt PVIs were defined as PCR or rapid antigen test-confirmed infections following at least two immunizations. Subclinical PVI were defined as a three-fold increase in anti-spike IgG BAU/mL occurring in the absence of concurrent vaccination and/or any increase in anti-spike IgG BAU/mL associated with nucleoprotein seroconversion (BAU/mL > 24.63). Timepoints following a clinically overt or subclinical PVI were excluded from vaccine-only IgG analysis.

### Statistical analysis

To estimate the longevity and decay of detectable circulating binding antibodies in sera collected from study participants, we applied nonlinear regression analysis for curve fitting. Both one-phase and two-phase decay curves were applied to datasets to determine which exponential model provided the best fit, assessed by Akaike’s Information Criterion (AIC) (alpha = 0.05). The rates of decay, antibody half-life, and time at which individuals remained above the 90%, 75%, and 50% protection levels were calculated from these best fit curve models. To estimate the longevity of the antibodies, we set a constraint in the curve model equal to anti-spike IgG concentration of 14.4 BAU/mL that we have established by receiver operator curve analysis as a threshold cutoff for seropositivity using serum samples from PCR-confirmed SARS-CoV-2-infected and non-infected individuals^20^. Decay rates were compared by constraining the rate constant parameter to be equal between curves, and using an F test to compare the fit of the constrained and unconstrained models.

## Supplementary information

Supplementary Information

## Data Availability

Data and key materials utilized in this study are available as a shareable resource upon reasonable request to the corresponding author.
