# Supplementary material for: Estimating the decay of protective antibodies induced by SARS-CoV-2 mRNA vaccination and hybrid immunity
Source: Npj Viruses. 2025 Oct 29;3:76. doi: 10.1038/s44298-025-00156-3 (PMC12572167; doi:10.1038/s44298-025-00156-3)

SUPPLEMENTARY INFORMATION

Supplementary Table 1. Comparison of one- vs. two-phase decay models for antibody decay curve fit

Model	One-phase decay AIC value	Two-phase decay AIC value
Post-two dose decay model[1]	17543	17526
Post-three dose decay model[2]	3882	3883
Booster-only decay model[1]	4452	4455
Hybrid immunity decay model[2]	4104	4108

Supplementary Figure 1. STROBE chart

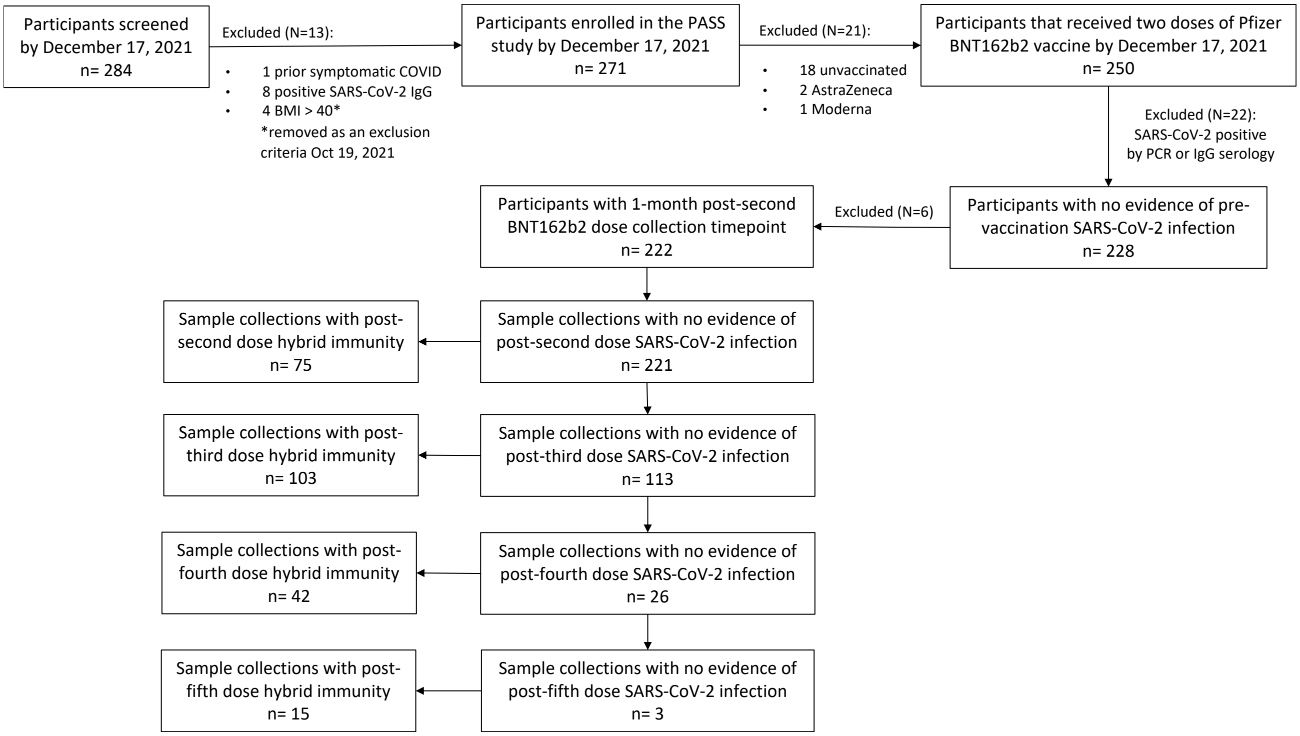

Supplementary Figure 2. Magnitude of SARS-CoV-2 antibody response improves after three doses. Anti-SC2 spike IgG measured in sera collected from participants one month-post receipt of two BNT162b2 doses (n=219) and three BNT162b2 doses (n=72). Statistical significance was assessed by Mann–Whitney U test, $p < 0.0001$.

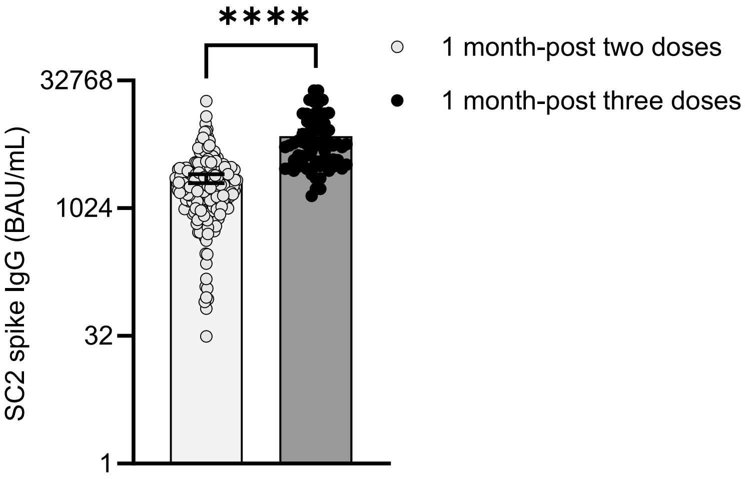

Supplementary Figure 3. Magnitude of SARS-CoV-2 antibody response improves after hybrid immunity. Anti-SC2 spike IgG measured in sera collected from participants one month-post vaccine-only immune boosting events (n=102) and one month-post PVI/hybrid immunity (n=107) were tested. Statistical significance was assessed by Mann–Whitney U test, $p < 0.01$.

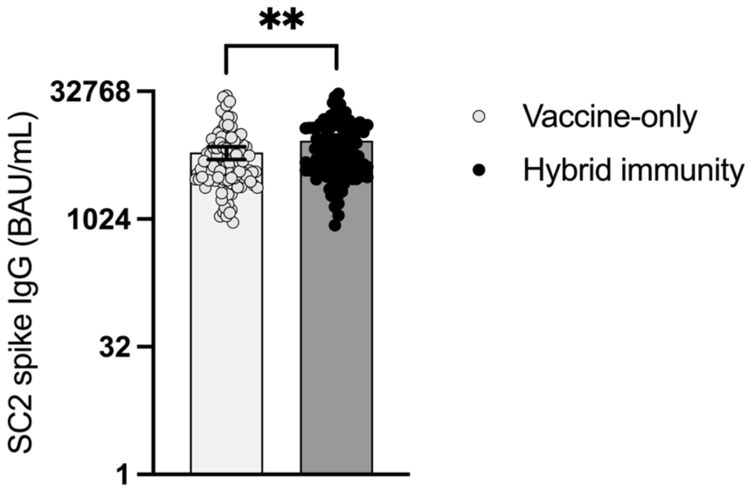

Supplement: Supplementary file 1 — Supplementary Information [file 44298_2025_156_MOESM1_ESM.pdf]
